# Evolution of human longevity: lessons from Hydra

**DOI:** 10.18632/aging.100510

**Published:** 2012-12-08

**Authors:** Almut Nebel, Thomas C. G. Bosch

**Affiliations:** ^1^ Institute of Clinical Molecular Biology, University of Kiel D-24105 Kiel, Germany; ^2^ Zoological Institute, University of Kiel D-24098 Kiel, Germany

Although the maximum human life span of 122 years is well established, the genetic and biochemical changes that influence the ability to reach old age in good physical and mental health are not very well understood. Both multiple environmental (~70%) and genetic (~30%) factors seem to play a role in attaining longevity [1]. So far, only a few genetic variants have been consistently reported to be associated with human longevity: the ε4 allele of the apolipoprotein E (APOE) gene, a mortality factor [1], and markers in the forkhead box O3A gene (FOXO3A) that show a modest beneficial effect upon survival in nonagenarians and centenarians [2]. Thus, FOXO3A can be considered the only confirmed longevity-enabling gene in humans to-date. However, it still remains to be elucidated through which mechanism(s) the as yet unidentified, underlying functional variation contributes to the longevity phenotype. FOXO3A is a key transcription factor in the insulin-IGF-1 (IIS) pathway, and in various model organisms FOXO3A homologues have been shown to be activated by caloric restriction or oxidative stress, resulting in increased life span. As the IIS pathway and its major players have been highly conserved throughout evolution, it is conceivable that also the human longevity-enabling FOXO3A variants could function via a similar stress-induced mechanism.

Clues to the role of FOXO3A in controlling longevity may be available through the comparative study of organisms which show no sign of aging. One of the very few examples of animals which appear to be truly immortal is the freshwater polyp Hydra [3]. Much of Hydra's remarkable immortality can be traced back to the asexual mode of reproduction by budding which requires a tissue consisting of stem cells with continuous self-renewal capacity [4]. Tissue function and maintenance in Hydra is based on three tissue-specific stem cell lineages which all have unlimited self-renewal capacity and can differentiate in one or more cell types [4]. Developing a transgenic method for Hydra cells [5] allowed tracing of GFP labelled cells and demonstrated that Hydra's stem cells indeed continuously proliferate and generate eternal lineages [5, 4]. How? This question has been plaguing some of us since the late 1980s.

With technological advances including the development of genomic resources [6] and novel computational tools not only the molecular signatures of the three stem cell lineages in Hydra have recently been uncovered [7], but also a new perspective was opened up on the molecular mechanisms controlling the unlimited self-renewal capacity of its stem cells. Intriguingly, the stem cell transcriptome signature showed that among the cell-intrinsic factors strongly expressed in all three stem cell lineages is FoxO. This finding immediately reminded us of several studies in worm, flies and humans [2] that showed FoxO expression levels and FoxO variants to be associated with longevity. Was it possible that FoxO is a key driver in Hydra stem cells? And could down-regulation of FoxO induce “aging” in Hydra? Gain-of-function and loss-of-function approaches have provided clear evidence that FoxO is important in the Hydra stem cell system [8]. Overexpression of FoxO in the multipotent interstitial stem cell lineage resulted in expression of germline marker nanos and vasa in terminally differentiated cells, indicating a soma-to-germline transformation. Silencing FoxO by antisense transcription had two interesting effects. It slowed down the proliferation of epithelial cells and it changed the expression pattern of antimicrobial peptides. In sum, Hydra's single FoxO gene was found to play a key role in controlling stem cell proliferation and terminal differentiation, suggesting an ancient role in maintaining developmental youth.

In the new study [8], a literally immortal model organism was induced to both stem cell senescence and immune senescence by altering the expression level of a single gene, the longevity factor FoxO. The data suggest that FoxO has ancient roles in controlling stem cell behavior that may underlie longevity. Hence, this FoxO regulation likely arose when multifunctional stem cells started to make differentiation decisions, which happened before the divergence of the Bilaterians more than 600 mio years ago. These new findings [8] extend the evolutionary reach of longevity factor FoxO to the early emerging metazoans.

The findings have captured the imagination of the popular press, and raised the skeptic's eyebrows. What lessons can actually be learned from the Hydra study? What does this mean for understanding human longevity? First, the Hydra results have moved the longevity-enabling FOXO3A gene from reported association to possible functions, corroborating and extending beyond previous observations in C. elegans and Drosophila. Second, the link between FoxO and components of the innate immune system [8] is of particular interest since aging processes in humans are known to result in impairment of both innate and adaptive immunity (“immunosenescence”) as well as in a pro-inflammatory status (“inflammaging”). Third, the Hydra study strengthens the earlier described role of FOXO3A in human stem cell maintenance and regulation. This hypothesis warrants further investigation and indicates another plausible mechanism through which FOXO3A variation may exert its effect on longevity. Attempts to extend the lessons learnt from Hydra to more complex organisms including humans will be challenging. However, the recent study is a proof of principle that investigations in Hydra stem cells hold promise. The more we learn about the role of FoxO in Hydra, the better we will understand how the gene and its variants contribute to longevity in humans.
